# Comparison of aneuploidy rate in spontaneous abortion chorionic villus between D6 and D5 thawed-frozen blastocyst transfer

**DOI:** 10.1186/s12884-023-05452-5

**Published:** 2023-02-28

**Authors:** Weie Zhao, Panyu Chen, Xiaoping Liu, Yujie Li, Xiaoyan Liang, Jingjie Li

**Affiliations:** Reproductive Medicine Research Center, Sixth Affiliated Hospital of Sun Yat-Sen University, 17#, Sogoulin Rd 510080, Guangzhou, 510655 China

**Keywords:** Blastocyst development speed, Frozen-thawed blastocyst transfer, Spontaneous abortion chorionic villus, Chromosome aneuploidy

## Abstract

**Background:**

To compare the aneuploidy rate in spontaneous abortion chorionic villus (SA-CV) after D5 and D6 thawed-frozen blastocyst transfer(TBT).

**Methods:**

This retrospective cohort study recruited 522 patients with early spontaneous abortion from March 2012 to January 2020 in the our center. The aneuploidy rate of SA-CV was compared according to the blastocyst development stage: D5 group (*n* = 398) and D6 group (*n* = 124).

**Results:**

Patients’ characteristics, including age, body mass index, follicle-stimulating hormone, fertilization methods, type of infertility, infertility duration, and gestational age when abortion, did not differ between the two groups (all *P* > 0.05). Although the mean number of embryos was significantly higher in D6 than in the D5 group (*P* < 0.001), the mean number of high-quality embryos was similar (*P* = 0.773). In the D5 group, 46.5% of SA-CV showed aneuploidy, which was comparable to 41.1% in the D6 group (*P* = 0.296). After further grouping according to age (> 35 years or ≤ 35 years), the difference between the D5 and D6 groups remained not statistically significant (*P* = 0.247 and *P* = 0.690). Multivariate logistic analysis showed that women’s age was independently associated with the aneuploidy rate (OR = 0.891; 95% CI: [0.854–0.930]; *P* < 0.001). The rate of chromosomal aneuploidy was significantly higher in the age > 35 years group than in the age ≤ 35 years group (61.0% vs. 39.4%, *P* < 0.001). Other factors, including blastocyst formation speed, were not significant predictors of aneuploidy rate.

**Conclusions:**

The rate of chromosomal aneuploidy in SB-CV after D6 TBT was comparable to that after D5 TBT. Chromosomal aneuploidy may not be a main factor contributing to the high prevalence early pregnancy loss at D6 group.

## Background

Blastocyst culture and transfer are effective in selecting optimal-quality embryos, improving endometrial synchronization, increasing cumulative pregnancy rates, and reducing multiple pregnancy rates. Normally, human embryos develop to the blastocyst stage on day 5(D5), however, some growth-retarded embryos require extended culture until day 6(D6)or day 7(D7) [1–4]. Meta-analysis showed that the speed of blastocyst development is related to blastocyst developmental potential and pregnancy outcomes in thawed blastocyst transfer (TBT) cycles, as evidenced by significantly lower clinical pregnancy and live birth rates and significantly higher miscarriage rates after D6 TBT compared to D5 TBT [1, 5]. These may be related to chromosomal abnormalities. A 10% increase in chromosomal aneuploidy rate has been reported in D6 blastocysts compared to D5 ones [6]. However, a few studies showed different findings in high-quality D5 and D6 blastocysts, with no significant difference in aneuploidy rates [7], and delayed blastocyst formation was not associated with chromosome abnormalities [8]. Thus, the opinion on the relationship between the speed of blastocyst formation and embryonic chromosomes is controversial.

To the best of our knowledge, all the above researches were based on studies of pre-implantation blastocysts. No data comparing the aneuploidy features in early spontaneous abortion chorionic villus(SA-CV) after D5 or D6 TBT, which is the direct evidence to confirm whether chromosomal abnormalities increase the risk of miscarriage after transfer of D6 blastocyst. Therefore, in this study, we compared the aneuploidy rate in abortuses between Day 5 and Day 6 groups, strengthening the understanding of the relationship between blastocyst development and chromosomal status.

## Methods

### Patients

**Fig. 1:**
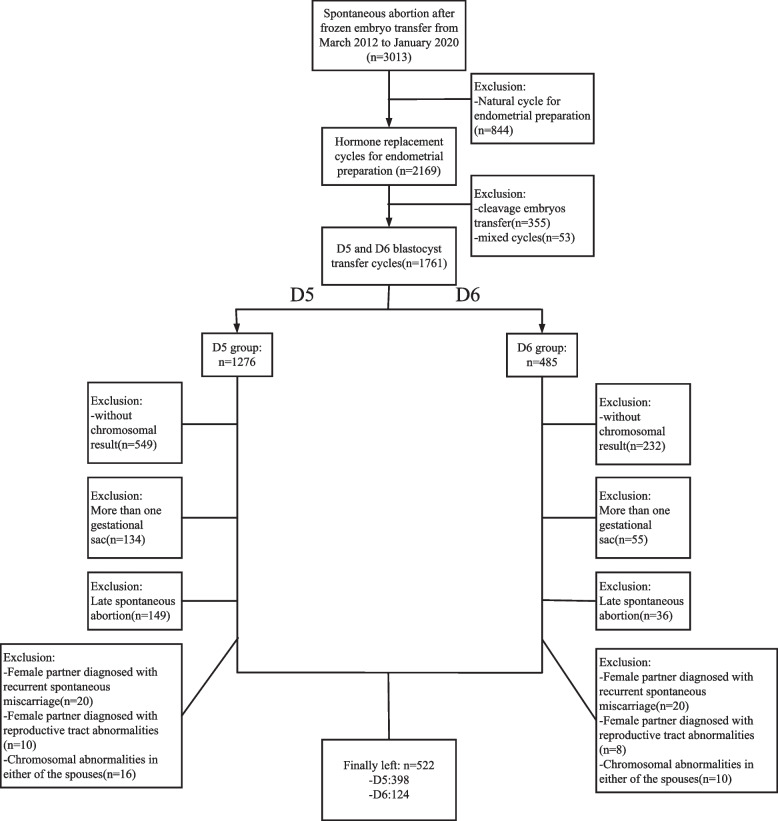
Flow chart of the study

This was a hospital-based retrospective study. The clinical data of patients who experienced pregnancy failure in the first trimester at the Sixth Affiliated Hospital of Sun Yat-sen University between March 2012 and January 2020 were analyzed. Pregnancy failure in the first trimester occurred at gestational age < 12 weeks and was diagnosed as follows: (1) Crown-rump length of ≥ 7 mm and no heartbeat, (2) mean sac diameter of ≥ 25 mm and no embryo, (3) absence of embryo with heartbeat ≥ 2 weeks after a scan that showed a gestational sac without a yolk sac, and (4) absence of embryo with heartbeat ≥ 11 days after a scan that showed a gestational sac with a yolk sac [9]. In this study, only TBT cycles with hormone replacement treatment (HRT) were included. They were divided into two groups according to the blastocyst developmental stage, D5 and D6. Exclusion criteria included twin or triplet pregnancies, chromosomal abnormalities in one or both partners, a history of miscarriages or repeated implantation failure, and reproductive tract abnormalities. The study population is shown in a flow chart (Fig. 1).Finally, a total of 522 patients were recruited, including 398 patients in the D5 group and 124 in the D6 group. The research protocol was approved by the ethics committee of the Sixth Affiliated Hospital of Sun Yat-sen University (2017ZSLYEC-016S). All patients signed an informed consent form before IVF treatment, allowing the use of their clinical information for scientific research.

### Controlled Ovarian Stimulation(COS),fertilization and embryos culture

GnRH agonist or GnRH antagonist regimen was chosen depending on the patients’ age, ovarian reserve, and previous COS response. The process has been described in detail in our previous study [10]. Conventional in vitro fertilization ( IVF) or intracytoplasmic sperm injection (ICSI) was performed according to sperm characteristics. The presence of two pronuclei was considered normal fertilization. The zygotes were cultured for 3 days and scored according to Cummins criteria [11]. Available D3 embryos (i.e. embryos with no less than 4 blastomeres and scored as level I-II)were cultured in a commercial sequential culture medium (G5 series; Vitrolife AB Company, Gothenburg, Sweden) to blastocyst stage and graded according to the criteria of Gardner and Schoolcraft [12]. If embryos on day 5 were at the stage of morula or early blastocyst, they would be cultured one more day. Only blastocysts at stages 3–6 with at least either inner cell mass or trophectoderm scored as grade B were vitrified for future transfer.

### Blastocysts vitrification, thawing and transfer

The vitrification of blastocyst was performed with a vitrification kit (Kitazato Company, Japan) according to the instruction manual. In TBT cycles, estradiol valerate (Bayer Pharmaceuticals, Germany) or Estradiol Gel (Oestrogel, Besins, Belgium) was administered on days 2–3 of menstruation. When the endometrial thickness was thicker than 8 mm and the duration of administration was at least 10 days, progesterone soft capsules (Utrogestan, Besins Healthcare, Belgium) were given vaginally for endometrium transforming, 1–2 blastocysts are thawed and transferred 5 days later.

### Chorionic villus culture and chromosome karyotyping

For patients diagnosed with pregnancy failure, vacuum curettage was performed, and chorionic villus samples were collected under sterile conditions. Villi cells were cultured in AmnioMAXTM-C100 basal medium with supplement (GIBCO, Grand Island, New York) and recovered and fixed when grown to appropriate density, then digested with trypsin solution and stained with Giemsa. Chromosome karyotype results were interpreted with reference to the international system of human cytogenetic nomenclature. At least 20 cells per sample were analyzed by an experienced cytogenetics technician. Karyotypes were analyzed for 50 cells if chromosomal aneuploidy was detected, and for 100 cells if mosaic karyotypes were detected.

### Outcomes

Aneuploidy was defined as a loss or gain of the genetic material of chromosome(s), including monosomies, trisomies, polyploidies, segmental aneuploidies, and complex chromosome aneuploidies. In this study, aneuploidy rate of of SA-CV was compered between D5 group and D6 group. Other factors such as age, type of infertility, diagnosed with PCOS were assessed to see if they affected chromosomal results.

### Statistical analysis

Statistical analyses were performed using IBM SPSS statistical software version 22 for Windows (IBM Corp., Armonk, NY, USA). The normality of continuous variables was examined by the Shapiro–Wilk test. The differences were statistically significant for the age at oocyte pick-up (OPU), body mass index (BMI), basal follicle-stimulating hormone (FSH), infertility duration, and gestational age when abortion. However, since the histograms and P-P plots indicated that the data distributions were not overly skewed, they were still expressed as mean (standard deviation,SD), and Student *t*-tests were performed for group comparisons. Other nonnormally distributed continuous variables were expressed as median(Q1-Q3) and Mann–Whitney U test was performed for group comparisons. Categorical variables were presented as percentages, and chi-square tests with Fisher’s exact test were used for frequency comparisons. Univariate regression analyses were performed for all variables to identify candidate factors. Variables showing a tendency to be associated with aneuploidy rate (*P* < 0.10) in the univariate analysis and literature-based confounders were selected for the multivariate model. *P* < 0.05 was considered statistically different.

## Results

Patient characteristics are shown in Table 1. There are no significant differences in age when OPU, BMI, FSH, type of infertility, fertilization methods, and gestation age when abortion between group D5 and D6 (all *P* > 0.05). The number of embryos transferred in the D5 group is significantly lower than that in the D6 group (*P* < 0.001), however, the difference is not significant in the number of high-quality embryos (*P* = 0.773).

**Table 1:** Patients’ characteristics in D5 group and D6 group

Variables	D5 group	D6 group	*P* value
Age when OPU(years)	32.6(4.8)	32.8(4.9)	0.657
Basal follicle stimulating hormone (IU/L)	6.6(2.2)	6.7(1.7)	0.569
Body mass index(Kg/m^2^)	22.1(3.5)	22.5(3.3)	0.312
Type of infertility			0.963
Primary infertility(%)	216(54.3)	67(54.0)	
Secondary infertility(%)	182(45.7)	57(46.0)	
Infertility duration(years)	4.8(3.3)	5.0(4.0)	0.622
No.of embryos transferred	1.0(1.0–1.0)	1.0(1.0–2.0)	< 0.001
No.of high- quality embryos transferred	1.0(1.0–1.0)	1.0(1.0–2.0)	0.965
Fertilization methods			0.182
IVF(%)	285(71.6)	81(65.3)	
ICSI (%)	113(28.4)	43(34.7)	
Diagnosed with PCOS (%)	69(17.3)	14(11.3)	0.108
Gestation age when abortion (weeks)	8.0(1.7)	8.0(1.7)	0.800

*OPU* Oocyte pick up, *IVF* In vitro fertilization, *ICSI *Intracytoplasmic sperm injection, *PCOS* Polycystic ovary syndrome

The chorionic chromosome aneuploidy rate in the D5 group is 46.5% (185/398), which is not significantly different from 41.1% (51/124) in the D6 group (*P* = 0.296) (Table 2). After excluding potential confounding factors such as age when OPU, infertility type, diagnosed with PCOS, gestational age, the difference in the rate of chromosomal aneuploidy between the D5 and D6 groups is still not significant (OR = 0.756; 95% CI: [0.429–1.220]; *P* = 0.225) (Table 3).

**Table 2:** Comparison of aneuploidy rate in the product of conception between D5 group and D6 group

Group	Total aneuploidy rate (%)	Aneuploidy rate of D5 group (%)	Aneuploidy rate of D6 group (%)	*P* value
All patients		185 (46.4)	51 (41.1)	0.296
≤ 35 years (%)	150 (39.4)	117 (41.1)	33 (34.4)	0.247
> 35 years (%)	86 (61.0)	68 (60.2)	18 (64.3)	0.690
*P* value	< 0.001	0.001	0.005

**Table 3:** Multivariable logistic regression analysis of potential factors association with aneuploidy rate in both D5 and D6 groups

	Univariate analysis		Multivariate analysis	
	OR(95% CI)	*P* value	OR(95% CI)	*P* value
Age when OPU (years)	0.878(0.844–0.914)	< 0.001	0.891(0.854–0.930)	< 0.001
Body mass index(Kg/m^2^)	0.973(0.925–1.023)	0.286		
Type of infertility	0.518(0.365–0.736)	< 0.001	0.779(0.530–1.146)	0.205
Infertility duration	0.964(0.917–1.013)	0.148		
Diagnosed with PCOS	0.531(0.323–0.871)	0.012	0.724(0.429–1.220)	0.225
Fertilization Mehtod (IVF/ICSI)^a^	0.880(0.604–1.282)	0.505		
No.of high quality embryos transferred	1.207(0.848–1.719)	0.296		
Blastocyst developmental stage(D5/D6)^b^	0.804(0.535–1.210)	0.296	0.756(0.491–1.162)	0.202
Abortion age (weeks)	0.927(0.833–1.031)	0.161	0.961(0.861–1.072)	0.473

*OPU* Oocyte pick up, *IVF* In vitro fertilization, *ICSI* Intracytoplasmic sperm injection, *PCOS* Polycystic ovary syndrome

^a^IVF as reference

^b^D5 as reference

Age when OPU significantly relate with chromosomal aneuploidy rates (OR = 0.891; 95% CI: [0.854–0.930]; *P* < 0.001). The rate of chromosomal aneuploidy is significantly higher in the age > 35 years group than in the age ≤ 35 years group (61.0% vs. 39.4%, *P* < 0.001). However, there is no significant difference in chromosomal aneuploidy rates between the D5 and D6 groups in both age > 35 years and age ≤ 35 years (*P* = 0.247 and *P* = 0.690) (Table 2).

## Discussion

The relationship between the pace of blastocyst development and chromosomal aberrations in villi has not been previously reported. Our results showed (Table 2) that the chromosome aneuploidy rate of SA-CV in the D6 group was comparable to that of the D5 group. This is inconsistent with the studies of pre-implantation blastocysts, which showed that the aneuploidy rate of D6 blastocysts was significantly higher than that of D5 blastocysts [6, 13, 14]. Possible reasons included maybe as following.

### Chromosomal aneuploidy between blastocyst TE and SA-CV manifest different characteristics

Although chromosomal aneuploidy is widely accepted as the most significant cause of spontaneous abortion in humans, different types of abnormality have different fates, manifesting with different characteristics from the blastocyst stage through early implantation to their ultimate fate. The main chromosomal abnormalities in oocytes and embryos were monosomy and trisomy [15, 16]. Large numbers of genes missing caused by monosomy are likely to lead to a lethal change in cellular pathways, such as massive methylation, transcriptomic, and proteomic changes. They may experience growth retardation or developmental failure and loss early due to severe developmental defects, as evidenced by failure in blastocysts forming and implantation or cessation of development after implantation (biochemical pregnancy) [17, 18]. In contrast, gaining a chromosome has relatively less impact than losing a chromosome [19]. Some particular trisomies are not lethal for blastocysts, they can continue to develop and successfully implant but still, abort at a certain stage. Only a few aneuploidies can result in live births, such as trisomy 21 and 45XO. The most notable change from blastocyst to SA-CV was an increased rate of trisomies, whereas the incidence of autosomes monosomies decreased by 30-fold [15]. Embryos with different chromosomal aneuploids arrest at different stages, therefore, we speculate that the chromosomal abnormalities affecting the speed of blastocyst development may be more severe, these embryos lost before or shortly after implantation, resulting in a lower clinical pregnancy rate (CPR) in the D6 group. However, there is no literature comparing the different distribution of chromosomal aneuploidy between D5 and D6 blastocysts. Further studies are needed to determine whether D6 blastocysts are with a higher rate of monosomy and other severe abnormalities than D5 blastocysts.

### Non-chromosomal factors interfering with pregnancy outcomes after D6 TBT

Lower CPR and LBR of euploidy D6 blastocysts than euploidy D5 blastocysts [20–22], indicated other factors that affect the blastocyst development and the reproductive potential. Such as the metabolic or epigenetic or organelle abnormalities of the embryo. The study by Wu et al. provided evidence that D5 blastocysts contained significantly greater mitochondrial DNA quantity than D6 blastocysts [23]. Hashimoto et al. have reported that spindle abnormalities are significantly higher in D6 blastocysts, which block cell mitosis and reduce cell number and development retardation [24].

Maternal age is an independent factor affecting pregnancy outcomes in both spontaneous and IVF pregnancies. LBR decreases with age increasing and is less than 1% in those older than 44 years [25].While the miscarriage rate shows the opposite trend and exceeds 50% in those older than 40 years. Chromosomal abnormalities are the main cause of poor age-related pregnancy outcomes. Oocytes/embryos aneuploidy rates are significantly higher in women of advanced age [26]. Older women transferring euploidy embryos have similar pregnancy outcomes to younger women [26]. Our results showed the same conclusion, with 61.0% of miscarriages caused by aneuploidy in women over 35 years of age, compared to a value of 39.4% in women under 35 years of age. The great majority of aneuploidy is the consequence of abnormal meiotic chromosome segregation during oocyte formation [16]. However, the rate of oocyte aneuploidy was not linearly related to age, but rather showed a U-shaped curve distribution, consistent with an inverse U-curve in humans' natural fertility, where young females (13–20 years) and women of advancing maternal age (AMA, the mid-30 s and above) show reduced fertility rates. Meiosis I nondisjunction (MI NDJ), in which gain or loss of a whole chromosome, decreased with female age. Precocious separation of sister chromatids (PSSC) or predivision, sister chromatids of one homolog separating in meiosis I, and reverse segregation (RS), when both homologous chromosomes split their two sister chromatids already at meiosis I, increased with female age. This combination of distinct age-dependent error types forms the U-curve of aneuploidy in human oocytes [27]. In our study, the minimum age of the patients was 23 years, so it was not possible to analyze the distribution of SA-CV aneuploidy in young females and women of AMA.

### Value of conventional karyotyping in early pregnancy loss

Aneuploidy is the most common numerical abnormality defined as a loss or gain of the genetic material of chromosome(s), the major types include monosomies, trisomies, polyploidies, segmental aneuploidies, and complex chromosome aneuploidies. It was reported that these abnormalities account for more than 87 ~ 93% of chromosomal abnormalities in SA-CV [28, 29]. Although copy number variants(CNVs) less than 3 Mb in euploid POCs distribute in all chromosomes, the clinical significance of these variants is unclear [30]. Therefore, karyotyping analysis is the main method of assessing chromosomal results in SA-CV.

Conventional karyotyping analysis can detect aneuploidy, balanced/unbalanced translocations, and inversions and is the first-line method for the diagnosis of chromosomal aberrations. However, the requirement for a large number of cells, long culture times and high failure rates, low resolution (> 5–10 Mb) limit its efficiency. Chromosomal microarray analysis (CMA) was shown to be superior to cytogenetics due to a significant increase in the test success rate [31]. It also could detect chromosomal duplication/deletion more than 200 Kb, uniparental disomy, loss of heterozygosity, and unbalanced translocation [31]. Nevertheless, the chromosomal abnormalities detected by CMA in early abortion are mainly numerical abnormalities and macro-segmental abnormalities(> 5 MB) [15, 28, 29], which can also be detected by conventional karyotyping. A meta-analysis also showed only a mean increment yield of 1% (95% CI, 1–2%) in CMA over conventional karyotyping in patients without recurrent pregnancy loss [31].

As a retrospective study, the present work has some limitations. However, we took measures to improve its credibility. (1) Only thaw-frozen cycles using HRT were included to minimize the influence of endometrial factors. (2) The underlying conditions of the included patients were controlled, such as excluding patients with miscarriage history or repeated implantation failure and excluding patients with chromosomal abnormalities. (3) All data in this study were from the same center. Experienced and fixed staffs were in charge of chorionic villus chromosome testing to reduce bias related to the operator. (4) A total of 522 patients were included in this work. The sample size was large enough to reflect the actual condition. Therefore, the results are credible to provide reference information on clinical treatment and provide guidance for patient counseling.

In conclusion, the rate of chromosomal aneuploidy in SB-CV after D6 TBT was comparable to that after D5 TBT, suggesting that chromosomal abnormalities may not be a factor contributing to the high prevalence early pregnancy loss in the D6 group. More studies are needed regarding the poorer pregnancy outcome in D6 than in D5 groups.

## Data Availability

The datasets generated during the current study are not publicly available since our institution prohibits public data sharing. However, the datasets are available from the corresponding author on reasonable request.

## Abbreviations

OPU: Oocyte pick up
IVF: In vitro Fertilization
ICSI: Intracytoplasmic sperm injection
SA-CV: Spontaneous abortion chorionic villus
TBT: Thawed-frozen blastocyst transfer
COS: Controlled ovarian stimulation
BMI: Body mass index
PCOS: Polycystic ovary syndrome
